# The Governance and Implementation of the National Action Plan on Antimicrobial Resistance in Tanzania: A Qualitative Study

**DOI:** 10.3390/antibiotics10030273

**Published:** 2021-03-09

**Authors:** Gasto Frumence, Leonard E. G. Mboera, Calvin Sindato, Bugwesa Z. Katale, Sharadhuli Kimera, Emmy Metta, Anna Durrance-Bagale, Anne-Sophie Jung, Stephen E. Mshana, Taane G. Clark, Mark Rweyemamu, Helena Legido-Quigley, Mecky I. N. Matee

**Affiliations:** 1 Muhimbili University of Health and Allied Sciences, Dar es Salaam 65001, Tanzania; emetta2000@gmail.com (E.M.); mateemecky@yahoo.com (M.I.N.M.); 2 Eastern and Southern Africa Centers of Excellence for Infectious Diseases of Humans and Animals (SACIDS-ACE), Morogoro 3019, Tanzania; lmboera@gmail.com (L.E.G.M.); csindato@gmail.com (C.S.); bugwesa2002@yahoo.co.uk (B.Z.K.); sikimera@gmail.com (S.K.); stephen72mshana@gmail.com (S.E.M.); mark.rweyemamu@btinternet.com (M.R.); 3 Tabora Research Centre, National Institute for Medical Research, Tabora 45026, Tanzania; 4 Tanzania Commission for Science and Technology, Dar es Salaam 4302, Tanzania; 5 Sokoine University of Agriculture, Morogoro 3019, Tanzania; 6 London School of Hygiene and Tropical Medicine, London WC1E 7HT, UK; anna.durrance-bagale@lshtm.ac.uk (A.D.-B.); Anne-Sophie.Jung@lshtm.ac.uk (A.-S.J.); taane.clark@lshtm.ac.uk (T.G.C.); ephhlq@nus.edu.sg (H.L.-Q.); 7 Catholic University of Health and Allied Sciences, Mwanza 33109, Tanzania; 8 SACIDS Foundation for One Health, Sokoine University of Agriculture, Morogoro 3019, Tanzania

**Keywords:** antibiotics, antimicrobial resistance, implementation plan, governance, Tanzania

## Abstract

Tanzania launched its first National Action Plan (NAP) on antimicrobial resistance (AMR) in 2017 to reduce the burden of AMR in the country and contribute to the global response. We aimed to analyze the implementation of the NAP on AMR in Tanzania using the governance framework. In-depth interviews were conducted with human and animal health practitioners and national-level policy actors. We adapted Chua’s AMR governance framework to analyze the development and implementation of the NAP in Tanzania. Implementation of the NAP has realized several achievements, including: (i) the establishment of a functioning Multi-Sectoral Coordinating Committee for coordinating the implementation of AMR activities; (ii) existence of governance structure; (iii) establishment of human and animal surveillance sites; (iv) creation of AMR awareness in the community and (v) availability of guidelines at the health facility level to ensure AMR stewardship. However, some dimensions of the governance areas, including reporting and feedback mechanisms, accountability, transparency and sustainability of AMR plans, are not effectively implemented. Addressing these challenges should involve strengthening the collaboration of the different sectors involved at different NAP implementation levels by careful planning and coordination, and provision of adequate resources to ensure sustainability.

## 1. Introduction

Antimicrobial resistance (AMR) is a global public health threat. Several studies have reported the causes of rising AMR, including self-medication [1,2], antibiotic overuse [3], clinicians’ over-prescription, a strong belief by the public in antibiotics, including non-prescription purchases [4], veterinary antibiotic use for prophylaxis and growth promotion [5] and inadequate knowledge on Infection prevention and control (IPC) and antimicrobial use and limited access to veterinary and extension services, paving the way for self-treatment and opportunism by profit-driven non-professional veterinary drug sellers and weak veterinary laws [6]. Furthermore, inappropriate antibiotic use, inadequate dosing and incomplete doses are some of the human malpractices that have contributed to the emergence and spread of antibiotic resistance [7]. It is projected that by 2030, AMR could force up to 24 million people into extreme poverty [8]. AMR disproportionally affects low-and middle-income countries (LMICs), characterized by relatively weak health systems [9], which are often without adequate effective tools for the prevention and treatment of drug-resistant infections [10]. Most of these countries have limited access to existing and new quality-assured antimicrobials. Recognizing this threat and in response to the Agenda of the 68th World Health Assembly (WHA) in May 2015, Tanzania launched its National Action Plan (NAP) on Antimicrobial Resistance 2017–2022 [11], to tackle AMR using a one health approach. This five-year plan outlines five key strategic objectives to address AMR. The development of the NAP was coordinated by the Ministry of Health, Community Development, Gender, Elderly and Children (herein after referred to as Ministry of Health, MoH) in collaboration with the Ministry of Livestock and Fisheries and the World Health Organization (WHO). Despite the fact that the NAP has been in place for more than three years, AMR is still a challenge for human, animal and environmental health in Tanzania [6,12,13].

In several LMICs, including Tanzania, implementation of NAPs for AMR remains fragmented and lacks comprehensive multi-sectoral and multi-pronged approaches [14]. Moreover, interventions, such as those focusing on improving public health systems and increasing access to clean water and sanitation, tend to focus disproportionately on urban geographies [15]. These constraints indicate that AMR is one of the most persistent public health challenges that requires a domestic governance framework to guide successful implementation of NAPs [14]. This study analyzed the implementation of the NAP in Tanzania using the governance framework to provide guidance and recommendations. Specifically, we used the framework to examine: (i) who was involved in developing the plan; (ii) what are the objectives Tanzania aims to achieve and how; (iii) its implementation (how are the identified NAP actions/activities implemented); and (iv) how are NAP activities monitored and evaluated.

### Conceptual Framework of Analysis

**Figure 1:**
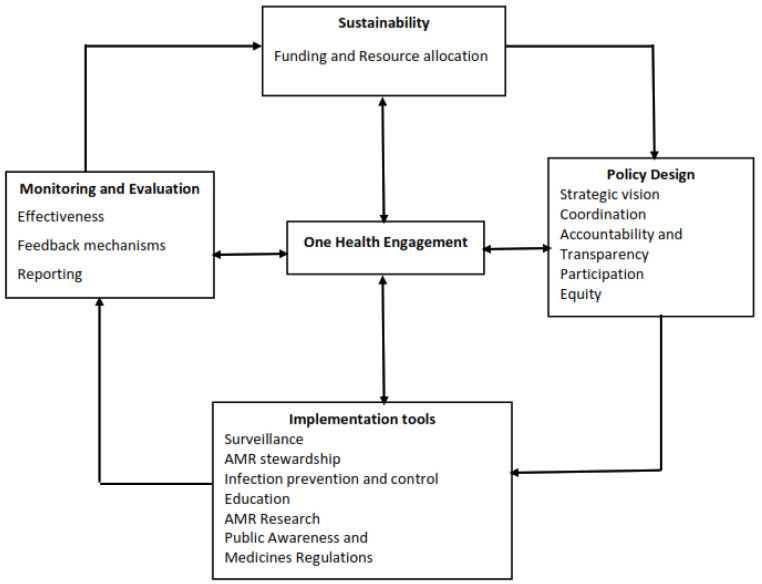
Antimicrobial resistance (AMR) governance framework with four areas and 15 domains adapted from Chua et al., 2021.

Despite widespread agreement that tackling AMR domestically can only be achieved through a strong governance framework, it is not clear how various efforts to implement the NAP in Tanzania are following the framework. We adapted Chua’s AMR governance framework to analyze the development and implementation of the NAP for combating AMR in Tanzania [16]. The framework (Figure 1) consists of five main governance areas: policy design, implementation tools, monitoring and evaluation, sustainability and one health engagement. Policy design focuses on general and procedural issues, such as broad participation in the development process, coordination across sectors and levels (from sub-national to national), transparency, implications of equity of AMR policies and accountability and coordination mechanisms on how to achieve NAP objectives. The implementation tools refer to various interventions for combating AMR, including antimicrobial stewardship programs, surveillance, infection prevention and control (IPC) measures, public awareness activities, medicine regulation and education of relevant professionals. The monitoring and evaluation component consists of reporting and feedback mechanisms, which allow regular review and evaluation of the NAP and its (cost-)effectiveness. In addition, this area assesses whether relevant policies and incentives are available to incentivize research and development of innovative antimicrobials and other potential alternatives. The sustainability focuses on resource allocation and availability of a budget for implementation of the NAP. The fifth governance area is one health engagement and is situated at the center of all governance areas, indicating the importance of the involvement of human, animal and environmental health sectors (one health approach) in the implementation of the NAP. As Chua [16] suggests, in this study, we treat one health engagement as a separate governance area, which is situated at the center of the governance framework (Figure 1) since it influences all other governance areas.

## 2. Results

The analysis of the implementation of the NAP adapted to Chua’s governance framework consists of five main governance areas: policy design, implementation tools, sustainability, monitoring and evaluation and one health engagement. In the next few sections, we detail our findings according to each governance area.

### 2.1. Policy Design

The policy design analysis focused on strategic vision, coordination, participation, accountability and transparency and equity.

#### 2.1.1. Strategic Vision

Strategic vision implies that the NAP contains clearly defined goals and objectives to direct interventions. The Tanzania NAP identifies ten priority actions and specific interventions for each activity with quantifiable targets [11]. The five strategic objectives are to create awareness and understanding of AMR through effective information, education and communication, which has two priority actions (awareness raising and risk communication and education); to strengthen the knowledge and evidence base through surveillance and research with three priority actions (surveillance system, laboratory capacity and research and development) and to reduce the incidence of infection through effective sanitation, hygiene and infection prevention measures with two priority actions (infection prevention and control in healthcare and health waste management systems). Other strategic objectives are to optimize the use of antimicrobial agents in human and animal health, which has two priority actions (regulatory framework for preservation of antimicrobial agents and antimicrobial stewardship programs); and to develop the economic case for sustainable investment that takes account of the needs of all countries and to increase investment in new medicines, diagnostic tools, vaccines and other interventions, which has one priority action of ensuring sustainability of antimicrobial resistance interventions [11].

#### 2.1.2. Coordination

In Tanzania, the Ministry of Health is the central coordinating ministry, while the Multi-Sectoral Coordinating Committee (MCC) is responsible for coordinating, facilitating and overseeing the implementation of all AMR activities. According to the Tanzania national AMR surveillance framework [17], the implementation of the NAP operations shall be managed by different ministries and institutions but monitored through the MCC whose main role is to advise, receive and approve surveillance plans and reports. The MCC is also responsible for overseeing, coordinating and evaluating all AMR surveillance-related activities in the food and agriculture sectors, mobilizing required resources and convening scientific meetings and seminars [17].

#### 2.1.3. Participation

The reviewed literature and interviewees indicate that with the exception of community members, who were not involved, all other key stakeholders were involved in the development of the NAP. These included ministries responsible for public health and animal health, universities and research institutions, regulatory authorities (Tanzania Medicines and Medical Devices), government agencies (Tanzania Veterinary Laboratory Agency, National Health Laboratory Quality Assurance and Training Centre); international organizations (WHO, Food and Agriculture Organization of the United Nations); professional councils (Pharmacy Council, Veterinary Council of Tanzania), representatives of health facilities (Muhimbili National Hospital and District Medical Officer) and the private sector (Pharmaceutical Wholesalers Association) [11].

Interviewees had varying views on the involvement of different sectors in the preparation of the AMR NAP. Some reported that key sectors like public health, livestock and fisheries were involved. They stated that because every key sector was involved, it was easy to outline their roles in the implementation of the plan.

Another respondent claimed that all sectors had equal influence during the preparation of the NAP:

“I think all sectors were influential because each sector is directly involved and each sector has advantages in the action plan because we have seen that the issue of bacteria resistance doesn’t concern one sector, it affects all sectors”.

(Key Informant (KI): scientist from National Laboratory).

Several interviewees raised concerns that not all sectors were involved in the preparation of the NAP. The environmental sector was not represented despite the fact that waste management is a substantial concern within the NAP. The health sector was reported as the dominant sector throughout the development process and was given priority compared to other sectors.

“Of course, when you talk about these issues involving more than one sector, the human health sector has often been taking a big part (okay)…, even in the plan it took a big part but at the end of the day it has to touch other sectors because they provide a response to human health. Human health is leading because it is a priority”.

(KI: MoH official)

Most of the key informants said categorically that community members, who are key in the implementation of the AMR NAP as consumers of antimicrobials, were not included in preparing the plan.

“There was no direct community involvement but the government prepared action plan. After the plan is ready the community will be informed and the community will be educated on what the plan is all about”.

(KI: a scientist from the National Laboratory)

Another respondent said:

“I’m not sure about the community involvement. In the sessions which I attended I did not see a participant specifically from the community”.

(KI: MoH official)

#### 2.1.4. Accountability and Transparency

To ensure accountability and transparency within the NAP, a clear governance structure under the supervision of MCC has been established [11]. The NAP has a clear budget and sources of funding for each identified activity for the 5-year plan and this information is in the public domain as the NAP document is available online. To ensure the overall goal of combating AMR is met, the MoH developed the National AMR Surveillance Framework [17], which provides guidelines on how different AMR surveillance sites should provide quarterly progress reports to various administrative units. However, the interviewees suggest that information about resources such as funding to support the implementation of AMR interventions, including surveillance, is not always publicly available.

Facility-level respondents reported that during their weekly clinical meetings, they get feedback about drug resistance.

“The guidelines have helped to us observe drug resistance among patients, … we get information through weekly reports, daily reports and monthly reports”.

(KI: health center respondent, Ilala)

#### 2.1.5. Equity

According to the NAP, the Medicines and Therapeutic Committee (MTC) in each health facility is mandated to ensure the rational use of medicines, including antimicrobials. The MTCs should monitor prescribing practices to ensure they adhere to the standard treatment, and ensure the availability of up-to-date important reference materials for medicine information including the Standard Treatment Guidelines and Essential Medicines List [11]. Health facility interviewees reported that every clinician is supposed to have the treatment guidelines when delivering health services to patients.

“What I know is that every clinician must have guidelines that he/she uses to provide treatment to patients, guidelines for all the diseases. So, when the clinician finds that a patient has used a certain medicine for a long time without getting better, then he moves a patient to another medicine which is stronger”.

(KI: health facility in-charge, Ilala)

Regarding equal access to medicines, including antimicrobials, interviewed health workers reported that the facility management attempts to ensure that all medicines required are available to all patients who need them.

“So, we came up with a strategy that a pharmacist should make sure we have all the required medicines. When there is shortage, we should take immediate actions to get alternative medicines for our patients”.

(KI: health worker, Ilala)

Tanzania also has the Standard Treatment Guidelines and National Essential Medicines List, which reflect the policy of the government of ensuring the availability of safe and efficacious essential medicines to all its citizens. This is a tool used to promote access to essential medicines to achieve maximum therapeutic benefit and optimize patient outcomes [18].

### 2.2. Implementation Tools

The NAP outlines six dimensions of interventions, which are understood as tools for implementation. These are surveillance, antimicrobial stewardship programs, IPC measures, education, AMR research, public awareness activities and medicine regulation. The following sections examine each intervention.

#### 2.2.1. Surveillance

The National AMR Surveillance Framework aims at generating evidence on the burden of AMR among priority pathogens isolated in hospitals from patients. The strategic objectives of this framework are aligned with the National AMR Action Plan based on national needs and priorities [17]. The framework provides guidelines on how AMR surveillance across the human and animal sectors should be conducted.

Eight and three surveillance sites for humans and animals, respectively, have been established in Tanzania. These surveillance sites consist of five zonal referral hospitals, one national hospital and three regional hospitals. The surveillance sites are coordinated by the National Public Health Laboratory. The Sokoine University of Agriculture and two Zonal Tanzania Veterinary Laboratory Agency’s centers in Mwanza and Arusha are the only surveillance sites for animals. The 2020 GLASS reports show that Tanzania did not submit data on any priority pathogens in 2019 because the country had recently begun surveillance, with the participation of a few laboratories [19]. This was also highlighted by several of the policy makers. Through surveillance sites, the emergence and prevalence of AMR can be detected and monitored and the driving factors for its spread and high-risk groups can be determined and thereby enable plans for effective AMR interventions [20].

#### 2.2.2. Antimicrobial Stewardship

Policy makers stated that in ensuring that there is responsible use of antimicrobials by healthcare professionals, and more specifically, selection of the most appropriate antimicrobials, duration, dose and route of administration, the MoH has distributed the guidelines to all health facilities, including dispensaries. The MoH provides education to health workers through on-the-job training programs. The government has also introduced the guidelines for Health Training Institutions to impart knowledge on AMR to both the instructors and students. The idea behind it is to ensure that when students graduate, they know and can implement these standards and guidelines. One of the key informants stated:

“We have now developed antimicrobial stewardship guidelines which will be released at any time”.

(KI: TMDA official)

#### 2.2.3. Infection Prevention and Control

Infection prevention and control (IPC) across human and animal sectors is one of the key priorities in the NAP. A number of activities have been identified, including supportive supervision to all health facilities by quality assurance teams. These also include facilitating the availability of IPC-related equipment, supplies and guidelines, establishing a functioning vaccination program for human and animal health and engaging communities in the implementation of personal hygiene and environmental sanitation [11]. National Laboratory staff reported that a working group is responsible for the supervision of disease prevention nationally. They visit hospitals to check whether health staff comply with IPC procedures like hand washing, and the effective use of antimicrobials, as well as hygiene and environmental sanitation. However, it was not clear to what extent these working groups are effective in ensuring healthcare workers adhere to IPC procedures.

“We already have a guideline and have begun to distribute it to various regions in preventing and controlling the infection. We are also conduct sensitization campaigns to create awareness on personal hygiene”.

(KI: Ministry of Health official)

#### 2.2.4. AMR Research

The NAP has prioritized AMR research and development activities, which include engaging relevant stakeholders to identify current gaps in knowledge and potential research areas, developing national research guidelines on AMR and undertaking research related to AMR. The literature showed that more research on AMR has been conducted after introduction of the NAP, indicative of the growing importance of AMR in both academic and policy arenas. The Tanzania national budget allocates 1% of the country’s GDP to finance research activities [21]. This level is similar to Kenya, which has a comparable GDP. However, lack of information regarding how much is allocated to AMR-specific research is an indication of a lack of clear strategies to achieve the identified AMR objectives and goals. Moreover, in reviewing AMR articles (published from 2018–2020) none has indicated receiving financial assistance from the government of Tanzania [6,12,13,22].

#### 2.2.5. Education and Public Awareness

Interviewees reported that there are ongoing education programs to train Health Training Institutes’ tutors on guidelines and standards regarding antimicrobial use (AMU) and AMR so that they can train their students. In turn, students also participate in educating the community about AMU and AMR:

“Community health education has increased and it is not only from the Ministry but also from individual and other stakeholders. For example, there are Pharmacy student’s association collaborating with others people to educate the public about AMR”.

(KI: TMDA official)

Different models have been used to create AMR awareness in the general public. These include local television and radio programs, and newspaper articles produced by different media houses. There are also regular trainings to ensure proper understanding and awareness of AMU and AMR among the media.

“There is a technical working group whose main task is to provide education on AMU and AMR to the general public and students organizes concerts and symposia and involves students who go around sensitizing the community on AMR”.

(KI: MoH official)

Participants reported that during Antibiotic Awareness Week every November, many awareness activities are conducted by different stakeholders, including the MoH. When there are public events such as the *Nane Nane* (Farmers’ Day) exhibitions, the government and other stakeholders participate in creating awareness of drug resistance in both animals and humans.

“Government officials go direct to farmers and livestock keepers to provide education about antibiotics resistance”.

(KI: Ministry of Livestock and Fisheries)

#### 2.2.6. Medicine Regulation

A list of medicines is used at all levels of the healthcare system to ensure appropriate use of antimicrobials. According to the NAP, Tanzania has legislation for the regulation of the quality, safety and efficacy of medicines including antimicrobial agents. The Tanzania Medicine and Medical Devices Authority is responsible for managing all drugs, including antibiotics, in the country. The interviewed policy makers reported that, although the medicine regulations may be in place, they may only be partially implemented. One of the main reasons for low levels of implementation is a lack of capacity within primary healthcare facilities (dispensaries, health centers and district hospitals). These facilities often do not have adequate financial and human resources, which results in a lack of diagnostic tools and laboratory equipment to perform culture and antimicrobial susceptibility testing, as recommended by the guidelines.

“We expect that no person will be prescribed antibiotics before performing culture and sensitivity in order to be sure the medication you are prescribing is going to work. However, we failed because some hospitals are not equipped. I also think district hospitals do not have the capacity”.

(KI: MoH official)

### 2.3. Monitoring and Evaluation

Monitoring and evaluation is an important governance area in the implementation of the NAP as it generates evidence to inform policy makers, planners and implementers whether the implementation of the NAP is effective or not. It also involves dissemination of information and reports to different stakeholders about the implementation of the NAP.

#### 2.3.1. Reporting

There is an established collaboration between the government of Tanzania and the WHO, and AMR data are transmitted to the international surveillance system through WHONET software. While the human health sector reports findings to GLASS on a monthly basis, interviewees pointed out some difficulties in accessing this information. According to the NAP, national-level data ought to be used to inform decision-making processes, however, the existing limited data on AMR are still a challenge in Tanzania.

#### 2.3.2. Feedback Mechanisms

Interviewees reported that efforts have been made to put in place AMR focal persons for human and animal sectors at the national, zonal, regional and lower levels who are responsible for conducting supervision of AMU and AMR and provide feedback to the national-level organizations. The national AMR focal persons have the additional responsibility of reporting on the prevalence and trends in AMR to GLASS. One of the key informants stated:

“The national level AMR focal persons do conduct supervision of the lower levels. This is because, currently, only the zonal levels are functioning. It will take time before the programme is rolled out nation-wide and to all levels”.

(KI: MCC member)

#### 2.3.3. Effectiveness of Monitoring and Evaluation

The effectiveness dimension requires that nations should put in place mechanisms to monitor and evaluate the effectiveness of various AMR interventions and specific policies. For instance, nations should strive to measure the effect of AMR on human and animal health [23]. The AMR NAP has a well-stipulated monitoring and evaluation (M&E) plan for measuring the effects of interventions. According to the M&E plan, a mid-term review, which was to be performed after 2 years (2019) to monitor the implementation of the NAP, was not conducted. The end of term evaluation is planned for 2021. The reviewed literature did not show evidence of efforts done to evaluate the efficacy or cost-effectiveness of specific policies or interventions. However, key informants reported that the AMR monitoring team is planning to conduct a situational analysis, which is expected to provide evidence regarding the effectiveness of some of the implemented interventions. A lack of M&E limits the country in learning from AMR implementation experiences and identifying barriers to progress and formulating workable solutions to strengthen implementation of sustainable AMR interventions.

### 2.4. Sustainability

#### Funding and Resource Allocation

Sustainability of the NAP depends much on the allocation of resources and a dedicated budget. However, interviewed policy makers reported that the MCC receives funds from development partners to implement activities identified in the NAP. Most of the interviewed policy makers raised a concern that the sustainability of AMR interventions is questionable because the majority of them receive funds from development partners.

“Another challenge is insufficient funds compared to existing activities. And if I look clearly, I see that this AMR is more of donor funded project than the government so when donors leave, the situation will be difficult”.

(KI: FAO representative to MCC)

### 2.5. One Health Engagement

In a tripartite approach, the FAO, OIE and WHO emphasize that addressing health risks at the human–animal–plant–ecosystem interfaces requires strong one health engagement [24]. AMR is the global public health threat that requires one health engagement whereby different actors and sectors, such as human and veterinary medicine, agriculture, the environment and consumers, should be involved in the development and implementation of the AMR NAP. Findings revealed that the development and implementation of the AMR NAP have involved more human and animal health sectors compared with the environmental sector, which jeopardizes the future achievement of the AMR NAP’s goals and objectives.

“The preparation of this plan was coordinated by the Ministry of Health, as I explained it was the issue of one health so we were together with the Ministry of Livestock and Fisheries”.

(KI: Ministry of Health official)

## 3. Discussion

Our study has revealed that the implementation of the AMR NAP has realized several achievements, including: (i) the establishment of a functioning Multi-Sectoral Coordinating Committee for coordinating, facilitating and overseeing the implementation of all AMR activities; (ii) existence of governance structure; (iii) establishment of surveillance sites for humans and animals; (iv) creation of AMR awareness in the community; and (v) availability of guidelines at the health facility level to ensure AMR stewardship. The analysis reveals that some of the dimensions of the governance areas, including reporting and feedback mechanisms to monitor the NAP progress, accountability, transparency, resource mobilization for research activities and sustainability of AMR plans, were not effectively implemented, which poses challenges towards effective implementation of the NAP. Resolving these challenges requires a systematic governance approach to successfully achieve the desired goals and objectives.

Tanzania’s AMR NAP 2017–2022 has a strategic vision to provide long-term direction. This implies that the NAP has clear goals and objectives to provide direction to interventions [11]. The strategic vision and objectives of the AMR NAP in Tanzania are in line with the WHO global action plan on AMR [25]. In Tanzania, the MCC was identified as a central organization responsible for coordinating, facilitating and overseeing the implementation of all AMR activities, including surveillance of AMR, mobilizing resources and M&E of the plan across sectors. However, the MCC is largely dependent on development partners’ support, which will certainly limit its sustainability [26].

Participation in the development and implementation of the AMR NAP is an important dimension, which increases ownership and acceptance of the plan, thus facilitating its implementation. The study findings reveal mixed results. On one hand, the formulation of the NAP engaged one health key stakeholders such as health, livestock and fisheries, although the health sector dominated the process. Unfortunately, the environmental sector, which is a critical actor, was not involved. It was noted that some of the sectors still work in silos which implies that there is a weak inter-sectoral collaboration and accountability system. Other sectors prefer to be accountable to their seniors who are in the same sector and they are hierarchically accountable to them for other responsibilities, making one health engagement a challenge, which limits effective implementation of the NAP. Notably, cross-sectoral coordination is problematic in LMICs because the governance structures and mechanisms to bridge sectors, not only within government but also with nongovernmental partners and programs, are fragmented or inflexible [27,28,29]. This observation has also been observed in Thailand where, although different stakeholders were involved in the preparation of the plan, ownership and participation in the implementation have been reported as inadequate [30]. In Kenya, coordination of AMR NAP implementation was reported as a challenge since it is difficult to coordinate all activities across key sectors: animal, human and environmental health [31]. Similarly, in Nepal, a separate multi-sectoral steering committee representing human and animal health sectors as well as other stakeholders has been formed to facilitate the coordination of AMR activities between sectors, provide guidance and advocate for policies [32].

In Tanzania, community members, who are key in the implementation of the AMR NAP as consumers of antimicrobials, were not included in preparing the NAP but were involved during the implementation of the plan, especially during awareness creation campaigns, which aim at informing the community on the effects of AMR. In Tanzania, different stakeholders participated in a series of community awareness-raising activities calling for responsible use of antibiotics in humans and animals in order to reduce the effects of antibiotic resistance. A similar dilemma was revealed in a systematic review of several European countries that indicated that citizens were expected to be recipients of awareness activities or education interventions stipulated in the AMR NAP documents, rather than having an active role in taking proactive measures, including those targeting the reduction of the need for antibiotics [33].

We report the existence of frameworks for providing regular reports on AMR surveillance data. However, the findings revealed that in Tanzania, data for funding of AMU/AMR surveillance are not always publicly available [17], which limits transparency. We noted that implementation of the NAP is largely donor dependent, and thus, the sustainability of interventions is questionable.

Our study reveals that despite the existence of standard treatment guidelines and Medicines and Therapeutic Committees that could have ensured equity in accessing antimicrobials, there is no evidence to suggest that the implementation of the NAP is equitable. Access to qualified service providers, including doctors, laboratory technicians and pharmacists, may partly limit equitable access to existing essential antimicrobials. In LMICs, unskilled health workers and limited laboratory facilities have been reported as among the factors contributing to the unnecessary prescription of antimicrobials, thus increasing the incidence of AMR [10,34,35].

Our findings highlight a lack of surveillance infrastructure and capability as most of the health facilities, especially in the rural areas, do not have well-equipped laboratory facilities with skilled personnel. Thus, laboratory infrastructures need to be strengthened to extend surveillance sites to more public and private healthcare facilities. Similar issues arise for AMR surveillance in the animal health sector. These challenges are not unique to Tanzania, but studies indicate that several African countries face similar obstacles [36,37]. Furthermore, the African region has not developed adequate capacity for AMR surveillance because many countries lack adequate strategies [37,38].

In Tanzania, IPC is one of the key priorities in the NAP and the government has put in place IPC-related equipment, supplies and guidelines for the implementation of personal hygiene and environmental sanitation. However, there are no documented studies showing the effectiveness of these efforts in Tanzania. Similarly, Ghana has a national policy on IPC [39], however, the uptake by both tertiary and secondary levels of healthcare facilities is not optimal [40,41] and in most cases, compliance with hand hygiene recommendations before and after patient contact has been reported to be low [42]. A recent study has reported that most (95%) countries in Sub Saharan Africa (SSA) do not have fully functional Water, Sanitation and Hygiene (WASH) or environmental health standards in place across all healthcare facilities, with only six countries with an animal IPC program [38].

The reviewed literature demonstrates contrary findings about AMR education and public awareness in most African countries. Notably, there is little continuing education on AMU for key stakeholders such as prescribers and livestock keepers, including poultry farmers [6]. In many cases, irrational prescription practices are also driven by subtle violation of medical ethics at the expense of economic and professional profiteering [43]. The implementation of different education programs for different stakeholders should be considered by different AMR NAPs as instrumental in ensuring access to and appropriate use of effective antimicrobials. As part of Tanzania’s efforts to strengthen the education of public and animal health professionals, the government engages in activities to raise public awareness and educate the wider population about the risks of AMR. However, it is still not clear the extent to which these different AMR awareness creation approaches have been effective in delivering the messages. Like in Tanzania, Ghana deployed similar approaches, particularly the use of media in creating AMR and AMU awareness in the general public. In addition, Ghana used civil society organizations to create awareness on AMU and AMR in communities, specifically targeting farmers’ groups and their associations [44].

Despite the existence of legislations and a list of medicines and pharmacopoeia books to ensure the quality, safety and efficacy of medicines and to guide the management of drugs, we noted weak enforcement of these guidelines and legislations, which has resulted in mismanagement of antibiotics in Tanzania. This includes the dispensing of antimicrobials by unauthorized people and their sale in the auction markets like any other commodity [12]. Most LMICs reported having legislation in place, however, issues of counterfeit medicine were among the main problems caused by factors such as the lack of enforcement of laws and regulations [38].

Our findings reveal that the reporting of AMR data in Tanzania is limited as there are no effective mechanisms that feed information to the various centers responsible for decision and policy making, hence limiting the monitoring and evaluation of the extent of AMR. Both GLASS and WHONET were identified as reporting systems used in Tanzania. A systematic review on AMR in the Central African Region highlighted that there is a paucity of data because of the poor reporting system, which is partly a result of the limited and poor surveillance system [45,46]. Tanzania has put in place AMR focal persons to supervise AMU and AMR and provide feedback to national-level organizations. However, the extent to which these focal persons at different organizational levels are effective in conducting supervision and providing feedback to the higher authorities is not clear. Other studies recommended that, given the absence of an effective reporting and feedback system, countries need proper audit and feedback systems to help strengthen Antimicrobial Stewardship (AMS) systems [47].

In Tanzania, the NAP includes a section on monitoring and evaluation to measure the effectiveness of interventions. The Interagency Coordination Group on AMR reported that it is difficult for poor countries to put in place a robust and accountable system for monitoring and evaluating NAPs because many of the countries lack systems and capacity and where systems are available and functional, changes in political power or in governance systems at different levels become a threat to their sustainability [27]. Like M&E, research is mentioned in the NAP as one of the pillars of combating AMR. However, it is not effectively operationalized. Findings from other studies have also reported that LMICs often lack adequate capacity to support research and development [48].

This is the first study in Tanzania assessing the implementation of AMR NAPs using a structured governance framework modified from Chua’s framework (2021) to better adapt to our study findings. The framework allowed a structured format of analysis of various dimensions of governance areas, which helped to provide Tanzania’s context-specific analysis of best practices regarding implementation of the NAP. However, our study has one limitation. Some important information, such as how many resources are allocated for carrying out the NAP’s activities, could not be obtained. This further speaks to issues of transparency and accountability and the lack thereof within the NAP, but also to wider debates on public policy making.

## 4. Materials and Methods

### 4.1. Study Design and Aims

This exploratory qualitative study analyses the implementation of the NAP on AMR in Tanzania using the governance framework. It involved in-depth interviews with animal and human health practitioners at a sub-national level and senior officials from human, animal and environmental sectors at the national level. A review of relevant documents, including guidelines, policies and national and international AMR reports, was conducted to complement information generated from primary data sources. The exploratory study design was considered appropriate for this study because it helped the researchers to conduct an in-depth exploration of views from different key informants at the facility, district and national levels.

### 4.2. Study Settings

Three districts, namely, Ilala, Kibaha and Kilosa were selected for the sub-national-level actors. Ilala is an urban area, densely populated, with informal housing, transport infrastructure, dump sites, agriculture, industrial commercial activities, fishing and sand mining. It is highly polluted by effluents from Msimbazi River tributaries originating from different sources, leakage of effluent from waste dumps, abattoirs and domestic wastewater from septic tanks and pit latrines that are used by about 85% of the city population. The selection of the district was based on the following factors: many different livelihood activities and environmental contamination by effluents and wastes from different sources [6]. Kibaha is characterized by large- and small-scale poultry and fish farming that is likely to involve antimicrobials. Kilosa district has a large population of pastoralists keeping cattle, sheep and goats, and are known to treat their animals frequently with antimicrobials [12].

### 4.3. Selection and Recruitment of Study Participants

We used a purposive sampling strategy to draw the key informants from each group of actors, namely, laboratory technicians, livestock officers, in-charges of health centrers and dispensaries, pharmaceutical assistants and dispensers in Ilala, Kibaha and Kilosa districts. We identified key informants (practitioners) through a mapping exercise and under the guidance of officials from the district and ward/village levels. The policy makers at the national level were purposely selected from the ministries responsible for human health, livestock and fisheries, food and agriculture. The other national key informants included members of the AMR MCC and some from the National Regulatory Authority and National Health Laboratory Quality Assurance and Training Centre. The national-level key informants were selected because of their roles in the preparation and supervision of the implementation of the national action plan for AMR.

### 4.4. Data Collection, Management and Analysis

We conducted 111 in-depth interviews with key informants from national, district and health facility levels. Interviews lasted 45 to 90 min and were run by 12 research assistants. All research assistants were trained according to Guidelines on Ethics for Health Research in Tanzania [49] and we ensured quality control by conducting regular reviews of collected data. All interviews were conducted in Kiswahili, transcribed verbatim and translated into English. We used a thematic data analysis approach, which applies both inductive and deductive reasoning [50]. Furthermore, two levels of an interpretive approach were used to analyze data: the first level involved viewing or experiencing the implementation of the NAP from the subjective perspectives of the study participants and the second level was to understand the meaning of the participants’ experiences in order to provide a “thick description” of the implementation of the NAP. All investigators collectively identified and validated emerging themes across a sample of transcripts before a line-by-line analysis was conducted using NVivo 12 qualitative data analysis software (QSR International Pty Ltd. Version 12, 2018). Emerging themes were identified and categorized into the four governance areas: policy design, implementation tools, sustainability and monitoring and evaluation. At the national level, a saturation point was reached at the 9th participant, while at the district and lower level, the saturation point was reached at 9, 36, 32 and 34 at the national level, in Ilala, Kibaha and Kilosa districts, respectively (Table 1).

### 4.5. Ethical Considerations

All study participants provided their informed consent for inclusion before they participated in the study and they were informed about anonymity and confidentiality issues, and that they could withdraw from the study at any time they wished. The Medical Research Coordinating Committee of the Tanzania National Institute for Medical Research approved the protocol (Ref. No. NIMR/HQ/R.8a/Vol IX/3147). The study was conducted following the principles of the Declaration of Helsinki.

## 5. Conclusions

This study uses a governance framework to analyze the development and implementation of Tanzania’s AMR NAP according to three governance areas: policy design, implementation tools and monitoring and evaluation. The NAP is an important first step in fighting the increase in the prevalence of AMR in the country. While the NAP outlines strong objectives, operationalizing and implementing them require careful planning with attention to detail to ensure Tanzania can achieve its goals. This analysis indicates that despite the availability of the NAP, there are challenges in its implementation. These include weak AMR surveillance, inadequate resources for plan implementation sustainability and a lack of funding for research and monitoring the progress. Strengthening the one health approach involving different NAP implementation levels, combined with careful planning with adequate allocation of resources, should be given top priority in policy and decision making for the effective implementation of the NAP.

## Figures and Tables

**Table 1:** Category and numbers of key informants.

Participants’ Category	National Level	Ilala	Kibaha	Kilosa
Ministry of Health, Community Development, Gender, Elderly and Children	7	-	-	-
Ministry of Livestock and Fisheries	1	-	-	-
Implementing Partner (FAO)	1	-	-	-
Environmental Officer from Ilala Municipality		1	-	-
Pharmacist and Pharmaceutical Assistants		4	3	2
Public and Private Health Facility Laboratory Technologists/Technicians		7	8	8
Livestock Field Officers		3	4	5
Agro-vets		4	5	6
In-charges of Health Facilities		9	6	8
Dispensers		8	6	5
**Total**	**9**	**36**	**32**	**34**

## Data Availability

In-depth interview guiding questions used to generate information for the study are attached.

## Abbreviations

AMU	Antimicrobial use
AMR	Antimicrobial resistance
FAO	Food and Agriculture Organization
GLASS	The Global Antimicrobial Resistance and Use Surveillance System
IPC	Infection prevention and control
KI	Key informant
NAP-AMR	National Action Plan on Antimicrobial Resistance
MCC	Multi-Sectoral Coordinating Committee
TMDA	Tanzania Medicines and Medical Devices Authority
WASH	Water sanitation and hygiene

## References

[1] Spellberg B., Gilbert D.N. (2014). The future of antibiotics and resistance: A tribute to a career of leadership by John Bartlett. Clin. Infect. Dis..

[2] Llor C., Bjerrum L. (2014). Antimicrobial resistance: Risk associated with antibiotic overuse and initiatives to reduce the problem. Ther. Adv. Drug Saf..

[3] Lipsitch M., Samore M.H. (2002). Antimicrobial use and antimicrobial resistance: A population perspective. Emerg. Infect. Dis..

[4] McNulty C.A.M., Boyle P., Nichols T., Clappison P., Davey P. (2007). The public’s attitudes to and compliance with antibiotics. J. Antimicrob. Chemother..

[5] Van Boeckel T.P., Brower C., Gilbert M., Grenfell B.T., Levin S.A., Robinson T.P., Teillant A., Laxminarayan R. (2015). Global trends in antimicrobial use in food animals. Proc. Natl. Acad. Sci. USA.

[6] Kimera Z.I., Frumence G., Mboera L.E.G., Rweyemamu M., Mshana S.E., Matee M.I.N. (2020). Assessment of drivers of antimicrobial use and resistance in poultry and domestic pig farming in the Msimbazi river basin in Tanzania. Antibiotics.

[7] Omolase C., Adeleke O., Afolabi A., Ofolabi O. (2011). Self medication amongst general outpatients in a Nigerian community hospital. Ann. Ibadan Postgrad. Med..

[8] World Bank (2016). Drug-Resistant Infections: A Threat to Our Economic Future. World Bank Rep..

[9] Tomson G., Vlad I. (2014). The need to look at antibiotic resistance from a health systems perspective. Ups. J. Med. Sci..

[10] Pokharel S., Raut S., Adhikari B. (2019). Tackling antimicrobial resistance in low-income and middle-income countries. BMJ Glob. Heal..

[11] United Republic of Tanzania (URT) (2017). Tanzania National Antimicrobial Resistance Action Plan.

[12] Sindato C., Mboera L.E.G., Katale B.Z., Frumence G., Kimera S., Clark T.G., Legido-Quigley H., Mshana S.E., Rweyemamu M.M., Matee M. (2020). Knowledge, attitudes and practices regarding antimicrobial use and resistance among communities of Ilala, Kilosa and Kibaha districts of Tanzania. Antimicrob. Resist. Infect. Control.

[13] Manyahi J., Kibwana U., Mgimba E., Majigo M. (2020). Multi-drug resistant bacteria predict mortality in bloodstream infection in a tertiary setting in Tanzania. PLoS ONE.

[14] Kakkar M., Chatterjee P., Chauhan A.S., Grace D., Lindahl J., Beeche A., Jing F., Chotinan S. (2018). Antimicrobial resistance in South East Asia: Time to ask the right questions. Glob. Health Action.

[15] The World Bank (2019). Pulling Together to Beat Superbugs Knowledge and Implementation Gaps in Addressing Antimicrobial Resistance.

[16] Chua A.Q., Verma M., Hsu L.Y., Legido-Quigley H. (2021). An analysis of national action plans on antimicrobial resistance in Southeast Asia using a governance framework approach. Lancet Reg. Health West. Pac..

[17] United Republic of Tanzania (2018). National Antimicrobial Resistance Surveillance Framework.

[18] United Republic of Tanzania (2013). Standard Treatment Guidelines and Essential Medicines List.

[19] World Health Organization (WHO) (2020). Global Antimicrobial Resistance and Use Surveillance System (GLASS) Report.

[20] World Health Organization (WHO) (2018). Surveillance and Monitoring for Antimicrobial Use and Resistance IACG Discussion Paper 1.

[21] United Republic of Tanzania (2018). COSTECH Rolling Strategic Plan: 2016/17–2020/2021.

[22] Horumpende P.G., Said S.H., Mazuguni F.S., Antony M.L., Kumburu H.H., Sonda T.B., Mwanziva C.E., Mshana S.E., Mmbaga B.T., Kajeguka D.C. (2018). Prevalence, determinants and knowledge of antibacterial self-medication: A cross sectional study in North-eastern Tanzania. PLoS ONE.

[23] Anderson M., Schulze K., Cassini A., Plachouras D., Mossialos E. (2019). A governance framework for development and assessment of national action plans on antimicrobial resistance. Lancet Infect. Dis..

[24] WHO, FAO, OIE (2016). Antimicrobial Resistance: A Manual for Developing National Action Plans.

[25] World Health Organization (2015). Global Action Plan on Antimicrobial Resistance.

[26] Acharya K.P., Karki S., Shrestha K., Kaphle K. (2019). One health approach in Nepal: Scope, opportunities and challenges. One Health.

[27] World Health Organization (WHO) (2018). Meeting the Challenge of Antimicrobial Resistance: From Communication to Collective Action.

[28] Kayunze K.A., Kiwara A., Lyamuya E., Kambarage D.M., Rushton J., Coker R., Kock R. (2014). Practice of one health approaches: Bridges and barriers in Tanzania. Onderstepoort J. Vet. Res..

[29] Mlozi M.R.S., Rumisha S.F., Mlacha T., Bwana V.M., Shayo E.H., Mayala B.K., Malima R.C., Mashoto K.O., Mboera L.E.G. (2015). Challenges and opportunities for implementing an intersectoral approach in malaria control in Tanzania. Tanzan. J. Health Res..

[30] Sommanustweechai A., Tangcharoensathien V., Malathum K., Sumpradit N., Janejai N., Jaroenpoj S. (2018). Implementing national strategies on antimicrobial resistance in Thailand: Potential challenges and solutions. Public Health.

[31] Ecumenical Pharmaceutical Network and ReAct Moving Beyond Antimicrobial Resistance (AMR) National Action Plans Development to Implementation. Proceedings of the Africa Annual Conference.

[32] Acharya K.P., Subramanya S.H., Lopes B.S. (2019). Combatting antimicrobial resistance in Nepal: The need for precision surveillance programmes and multi-sectoral partnership. JAC Antimicrob. Resist..

[33] Castro-Sánchez E., Iwami M., Ahmad R., Atun R., Holmes A.H. (2018). Articulating citizen participation in national anti-microbial resistance plans: A comparison of European countries. Eur. J. Public Health.

[34] Munga M.A., Mæstad O. (2009). Measuring inequalities in the distribution of health workers: The case of Tanzania. Hum. Resour. Health.

[35] Shemdoe A., Mbaruku G., Dillip A., Bradley S., William J.J., Wason D., Hildon Z.J.L. (2016). Explaining retention of healthcare workers in Tanzania: Moving on, coming to “look, see and go”, or stay?. Hum. Resour. Health.

[36] Kariuki S., Dougan G. (2014). Antibacterial resistance in sub-Saharan Africa: An underestimated emergency. Ann. N. Y. Acad. Sci..

[37] Ndihokubwayo J.B., Yahaya A.A., Desta A.T., Ki-Zerbo G., Odei E.A., Keita B., Pana A.P., Nkhoma W. (2013). Antimicrobial Resistance in the African Region: Issues, Challenges and Actions Proposed. Key Determinants for the African Region.

[38] Elton L., Thomason M.J., Tembo J., Velavan T.P., Pallerla S.R., Arruda L.B., Vairo F., Montaldo C., Ntoumi F., Abdel Hamid M.M. (2020). Antimicrobial resistance preparedness in sub-Saharan African countries. Antimicrob. Resist. Infect. Control.

[39] Ministry of Health, Ghana (2015). National Policy and Guidelines for Infection Prevention and Control in Health Care Settings.

[40] Yevutsey S.K., Buabeng K.O., Aikins M., Anto B.P., Biritwum R.B., Frimodt-Møller N., Gyansa-Lutterodt M. (2017). Situational analysis of antibiotic use and resistance in Ghana: Policy and regulation. BMC Public Health.

[41] Labi A.K., Obeng-Nkrumah N., Bjerrum S., Aryee N.A.A., Ofori-Adjei Y.A., Yawson A.E., Newman M.J. (2018). Physicians knowledge, attitudes, and perceptions concerning antibiotic resistance: A survey in a Ghanaian tertiary care hospital. BMC Health Serv. Res..

[42] Asare A., Enweronu-Laryea C.C., Newman M.J. (2009). Hand hygiene practices in a neonatal intensive care unit in Ghana. J. Infect. Dev. Ctries..

[43] Basu S., Garg S. (2018). Journal of Medical Ethics and History of Medicine Letter Antibiotic prescribing behavior among physicians: Ethical challenges in resource-poor settings. J. Med. Ethics Hist. Med..

[44] Opintan J.A. (2018). Leveraging donor support to develop a national antimicrobial resistance policy and action plan: Ghana’s success story. Afr. J. Lab. Med..

[45] Njukeng P.A., Ako-Arrey D.E., Amin E.T., Njumkeng C., Wirsiy F.S. (2019). Antimicrobial Resistance in the Central African Region: A Review. J. Environ. Sci. Public Health.

[46] Mathew P., Jaguga C., Mpundu M., Chandy S.J. (2020). Building knowledge and evidence base on antimicrobial resistance in Africa, through ‘One Health’ based surveillance. Clin. Epidemiol. Glob. Health.

[47] Storr J., Twyman A., Zingg W., Damani N., Kilpatrick C., Reilly J., Price L., Egger M., Grayson M.L., Kelley E. (2017). Core components for effective infection prevention and control programmes: New WHO evidence-based recommendations. Antimicrob. Resist. Infect. Control.

[48] Seale A.C., Gordon N.C., Islam J., Peacock S.J., Scott J.A.G. (2017). AMR surveillance in low and middle-income settings-A roadmap for participation in the Global Antimicrobial Surveillance System (GLASS). Wellcome Open Res..

[49] Tanzania National Health Research Ethics Committee (2009). Guidelines of Ethics for Health Research in Tanzania.

[50] Fereday J., Muir-Cochrane E. (2006). Demonstrating rigor using thematic analysis: A hybrid approach of inductive and deductive coding and theme development. Int. J. Qual. Methods.

